# A screening strategy for the discovery of drugs that reduce C/EBPβ-LIP translation with potential calorie restriction mimetic properties

**DOI:** 10.1038/srep42603

**Published:** 2017-02-15

**Authors:** Mohamad A. Zaini, Christine Müller, Tobias Ackermann, Jeanette Reinshagen, Gertrud Kortman, Ole Pless, Cornelis F. Calkhoven

**Affiliations:** 1 European Research Institute for the Biology of Ageing (ERIBA), University Medical Center Groningen, University of Groningen, 9700 AD Groningen, The Netherlands; 2 Fraunhofer IME ScreeningPort, D-22525 Hamburg, Germany

## Abstract

An important part of the beneficial effects of calorie restriction (CR) on healthspan and lifespan is mediated through regulation of protein synthesis that is under control of the mechanistic target of rapamycin complex 1 (mTORC1). As one of its activities, mTORC1 stimulates translation into the metabolic transcription factor CCAAT/Enhancer Binding Protein β (C/EBPβ) isoform Liver-specific Inhibitory Protein (LIP). Regulation of LIP expression strictly depends on a translation re-initiation event that requires a conserved *cis*-regulatory upstream open reading frame (uORF) in the C/EBPβ-mRNA. We showed before that suppression of LIP in mice, reflecting reduced mTORC1-signaling at the C/EBPβ level, results in CR-type of metabolic improvements. Hence, we aim to find possibilities to pharmacologically down-regulate LIP in order to induce CR-mimetic effects. We engineered a luciferase-based cellular reporter system that acts as a surrogate for C/EBPβ-mRNA translation, emulating uORF-dependent C/EBPβ-LIP expression under different translational conditions. By using the reporter system in a high-throughput screening (HTS) strategy we identified drugs that reduce LIP. The drug Adefovir Dipivoxil passed all counter assays and increases fatty acid β-oxidation in a hepatoma cell line in a LIP-dependent manner. Therefore, these drugs that suppress translation into LIP potentially exhibit CR-mimetic properties.

Calorie restriction (CR; also called dietary restriction) without malnutrition increases health and lifespan in virtually all species studied^1^. Reduced signaling through the nutrient sensitive mechanistic target of rapamycin complex 1 (mTORC1) is thought to mediate many of the beneficial effects of CR^2^,^3^,^4^. In addition to CR, inhibiting mTORC1 either by pharmacological treatment, mutations, or low protein, high carbohydrate (LPHC) diets has similar beneficial effects^4^,^5^,^6^,^7^,^8^. A prominent result of CR or low mTORC1 signaling is a decrease in cancer incidence, which maybe related to the alterations in mTORC1-controlled metabolism^2^,^9^.

mTORC1 signaling coordinates the regulation of global protein synthesis and autophagy to adapt protein homeostasis to changing nutrient availability and growth factor signaling^10^. In addition, the expression of a subset of proteins is specifically regulated by mTORC1 through distinctive translation of their mRNAs involving *cis*-regulatory elements that make them responsive to regulators of translation. Previously, we presented that specific translation into the C/EBPβ protein isoform LIP (Liver-specific Inhibitory Protein) is under control of mTORC1 through regulation of the downstream eukaryotic translation initiation factor 4E (eIF-4E) binding proteins (4E-BPs), and that mTORC1-inhibition by rapamycin reduces LIP expression^11^,^12^. Translation of the C/EBPβ-mRNA involves two separate translation mechanisms, initiation and re-initiation: (1) Synthesis of the isoforms LAP and LAP* (Liver-specific Activating Protein) is the result of regular translation initiation where ribosomes scan the mRNA from the 5′-end to the first AUG-codon in a favorable Kozak sequence context to initiate translation (LAP* is often weakly expressed because it has no Kozak sequence) (Supplementary Fig. S1a and b); (2) translation into LIP requires the initial translation of a *cis*-regulatory upstream open reading frame (uORF) in the mRNA leader sequence, followed by the continuation of mRNA scanning and translation re-initiation from the downstream LIP-AUG codon (Supplementary Fig. S1c)^11^. Activated mTORC1 signaling stimulates the latter re-initiation into LIP but only marginally affects the initiation into LAP. The dependence of LIP expression on the presence of the uORF can be explained by the finding that translation of the uORF prevents translation initiation at the LAP initiation codon. Mechanistically this is based on the fact that uORF-post-termination ribosomes have to be reloaded with new initiator tRNA (Met-tRNA_i_^Met^) in order to perform a second translation re-initiation at the same mRNA molecule. The LAP initiation codon is too close (4 nt) to the uORF for the post-termination ribosomes to be reloaded with new Met-tRNA_i_^Met^ in time. Therefore, they omit the LAP initiation codon but can be reloaded with Met-tRNA_i_^Met^ early enough to re-initiate translation at the downstream LIP initiation codon (Supplementary Fig. S1c). The sophisticated structure of the C/EBPβ-mRNA renders its translation responsive to changes in availability or activity of translation-regulatory factors.

Met-tRNA_i_^Met^ is delivered to the ribosomes in a ternary complex with eIF2 and GTP and initiation is accompanied by GTP-hydrolysis followed by the release of the eIF2-GDP complex. The activity of eIF2 is restored by the guanine-nucleotide exchange factor eIF-2B, a process that is inhibited by eIF2α-kinases that phosphorylate eIF2α-subunit of eIF2-GDP and thereby limit translation under various stress conditions^13^,^14^. Since re-initiation at the LIP-AUG requires loading of a new initiator tRNA, re-initiation at the LIP-AUG is sensitive to eIF2α-kinases^11^.

While LAP/LAP* is a transcriptional activator LIP lacks the transactivation domains and can therefore act as a competitive inhibitor of LAP function^15^. Consequently, the ratio between LAP and LIP is crucial for the biological functions elicited by C/EBPβ. Genetic elimination of the uORF in mice abrogates regulated translation into LIP^12^,^16^. These C/EBPβ^ΔuORF^ knockin mice display a CR-type improved metabolic profile, including reduced fat accumulation and increased fatty acid β-oxidation, improved insulin sensitivity and glucose tolerance, and enhanced activity^12^,^17^. Intriguingly, these metabolic improvements are achieved without reducing calorie intake. Furthermore, genetically modified mice with mono-allelic or bi-allelic overexpression of LIP display an increase in cancer incidence^18^. Thus, pharmacological targeting of C/EBPβ-LIP expression may provide a promising strategy to screen for drugs with CR-mimetic properties for the treatment of metabolic disease and cancer.

Other transcripts than C/EBPβ use the mechanism of uORF-dependent re-initiation for translationally controlled protein expression, including the well-studied yeast Gcn-4 or the eukaryotic ATF-4 mRNAs as well as other mRNAs involved in proliferation and differentiation^19^. It is believed that post-uORF re-initiation involves the same translation factors as regular initiation. There is, however, emerging evidence that uORF-containing transcripts are in addition coordinately regulated by non-canonical re-initiation factors. This model was recently strengthened by the discovery that the complex of density regulated protein (DENR) and Multiple Copies in T-cell Leukemia (MCT-1) is a selective regulator of re-initiation involved in tissue growth^20^. In addition, our recent work suggests that proper re-initiation is disturbed by mutations in the ribosomal maturation factor gene Shwachman-Bodian-Diamond Syndrome (*SBDS*) that cause Shwachman Diamond Syndrome (SDS)^21^. Therefore, beyond the regulation of C/EBPβ translation per se coordinated uORF-dependent regulation of subsets of transcripts may be involved in controlling physiological as well as pathological alterations.

Conditions that reduce LIP levels like CR or other means of mTORC1 inhibition are explored for its feasibility to improve metabolic health and to treat cancer. Long term CR, however, is not manageable for most people and current treatment with mTORC1 inhibitors are of limited success and come with serious side effects^22^,^23^,^24^,^25^. Transcription factor function is considered to be largely undruggable and in case of LIP it would almost inevitably mean that LAP is targeted too, since LAP includes all sequences that make up LIP. Fortunately, we know that the specific translation mechanism that is responsible for LIP translation is potentially druggable, as reduction of LIP through pharmacological inhibition of mTORC1 exemplifies. Therefore, we set out to develop a screening strategy for the identification of compounds that alter C/EBPβ-uORF dependent translation re-initiation into LIP and thereby ultimately identify compounds that have CR-mimetic and/or anti-cancer activities. Here we present a cellular system containing C/EBPβ-uORF controlled luciferase-reporters that measures simultaneously translation initiation and re-initiation and is suited for high-throughput screening strategies. The screening system was validated with pharmacological and genetic approaches and used to screen a library of FDA approved drugs. Hereby we identified drugs known to inhibit mTORC1 as well as other drugs previously unknown to affect re-initiation/initiation ratios. Finally, a single drug was identified that reduces translation re-initiation in different cell lines, reduces endogenous LIP expression and increases β-oxidation in a mouse hepatoma cell line. Therefore, with our reporter we present a reliable tool for the search for small molecule compounds that may mimic calorie restriction in which C/EBPβ and other uORF controlled genes are causally implicated.

## Results

### A reporter system to emulate (monitor) C/EBPβ-mRNA translation

**Figure 1:**
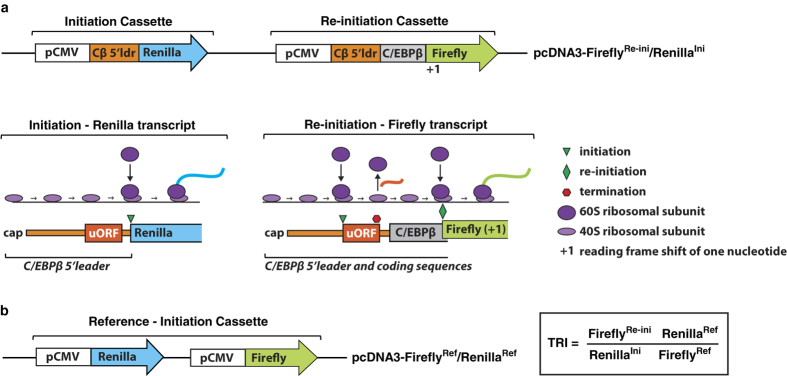
Translation Re-initiation Index (TRI) determination. (**a**) Representation of pcDNA3-Firefly^Re-ini^/Renilla^Ini^ plasmid containing the initiation and re-initiation cassette as indicated. Renilla luciferase (Renilla^Ini^) can only be translated by ribosomes that have scanned over the uORF in the C/EBPβ 5′leader sequence (Cβ 5′ldr) and directly initiate at the LAP-initiation AUG-codon that runs into the renilla reading frame, representing LAP translation. Translation of the firefly luciferase (Firefly^Re-ini^) is only possible by ribosomes that have first translated the uORF in the C/EBPβ 5′leader sequence (Cβ 5′ldr), resume scanning and re-initiate at the LIP-initiation AUG-codon of the firefly reading frame, representing LIP translation. The ribosomes that bypass the uORF and start translating the C/EBPβ-LAP reading frame (grey) cannot initiate translation of the downstream firefly reading frame because this is shifted +1 and therefore does not produce a luciferase signal. (**b**) Representation of the pcDNA3-Firefly^Ref^/Renilla^Ref^ plasmid that is devoid of C/EBPβ sequences and is used for measurement of reference translation levels of firefly luciferase (blue) and renilla luciferase (green). The formula used for calculation of the translation re-initiation index (TRI) is shown framed.

In order to identify small molecule compounds that modulate C/EBPβ-uORF dependent and differential translation of the C/EBPβ-mRNA into the protein isoforms LAP and LIP we designed a reporter system as a surrogate for this process. A single dual reporter plasmid was constructed that expresses a renilla luciferase transcript for the simulation of translation initiation into LAP and a firefly luciferase transcript for the simulation of translation re-initiation into LIP, both under control of the C/EBPβ-5′-leader sequence (Fig. 1a, pcDNA-Firefly^Re-ini^/Renilla^Ini^). Comparable to translation into LAP from the genuine C/EBPβ-mRNA, regular translation initiation after leaky scanning over the uORF results in expression of renilla luciferase from the initiation cassette (Renilla^Ini^) (Fig. 1a, Initiation Cassette). This cassette does not contain the downstream LIP-AUG and therefore uORF translation and eventual subsequent downstream re-initiation events are not detected. The efficiency of uORF-dependent re-initiation, simulating translation into LIP, is measured as expression levels of firefly luciferase from the re-initiation cassette (Firefly^Re-ini^) (Fig. 1a, Re-initiation Cassette). Ribosomes omitting translation of the uORF in the re-initiation cassette initiate translation at the proximal ORF consisting of C/EBPβ sequences that would mimic translation at the LAP initiation site (Fig. 1a, grey box), however, its product is not detected because the renilla reading frame is shifted with +1. In this way, only re-initiation is measured and direct initiation is left unnoticed. Alterations in the ratio between re-initiation and initiation are depicted as Firefly^Re-ini^/Renilla^Ini^ values for the compound library screens.

An additional reporter plasmid was constructed for measurement of reference levels of firefly (Firefly^Ref^) and renilla (Renilla^Ref^) luciferase through unrestrained translation (Fig. 1b, pcDNA-Firefly^Ref^/Renilla^Ref^). This reporter is used for the counter assay to control for effects not related to re-initiation/initiation efficiency like the rate of transcription, mRNA stabilization, general effects on translation or direct interference with the luciferase activity. Using measurements obtained with the pcDNA3-Firefly^Re-ini^/Renilla^Ini^ and the pcDNA3-Firefly^Ref^/Renilla^Ref^ plasmids a translation re-initiation index (TRI) can be calculated by using the formula TRI = Firefly^Re-ini^ Renilla^Ref^/Renilla^Ini^ Firefly^Ref^ (Fig. 1b, boxed formula). Hence, an TRI >1 indicates enhanced re-initiation and an TRI <1 indicates reduced re-initiation, respectively. TRI provides a more stringent normalized measurement for the validation of compounds identified by Firefly^Re-ini^/Renilla^Ini^ reporter system.

### Validation of the reporter system

**Figure 2:**
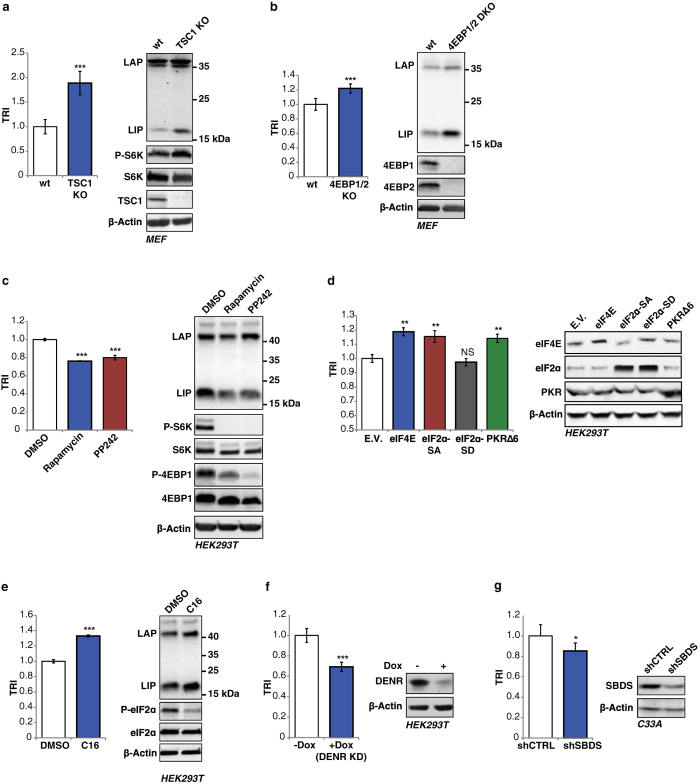
Reporter system validation. (**a**) Mouse embryonic fibroblasts (MEFs) that are deficient in the mTORC1-inhibitor protein TSC1 (TSC1-KO) or wt MEFs transiently transfected with pcDNA3-Firefly^Re-ini^/Renilla^Ini^ and pcDNA-Firefly^Ref^/Renilla^Ref^ reporter plasmids. The bar graphs show the calculated TRI mean values (n = 5). Immunoblots show the levels of C/EBPβ, TSC1, phospho-S6K, S6K and the β-actin loading control. (**b**) 4E-BP1/2 double knockout (DKO) MEFs and wt MEFs transiently transfected with reporter constructs as described in (**a**). The bar graphs show the calculated TRI mean values (n = 5). Immunoblots show the levels of C/EBPβ, 4E-BP1, 4E-BP2 and the β-actin loading control. (**c**) HEK293T cells stably expressing pcDNA3-Firefly^Re-ini^/Renilla^Ini^ or pcDNA-Firefly^Ref^/Renilla^Ref^ reporters treated with 200 nM rapamycin or 10 nM PP242 for 8 h. The bar graphs show the calculated TRI mean values (n = 3). The immunoblots show mTORC1 inhibition by reduced phosphorylation of 4E-BP1 and S6K in addition to β-actin as loading control. (**d**) HEK293T cells described in (**c**) transfected with eIF expression vectors. The bar graphs show the calculated TRI mean values (n = 3). Immunoblots show levels of eIF4, eIF2α, PKR and the β-actin loading control. (**e**) HEK293T cells described in (**c**) treated with the PKR inhibitor (C16) for 8 h. The bar graphs show the calculated TRI mean values (n = 3). Immunoblots shows the reduced phosphorylation of eIF2α and the β-actin loading control. (**f**) DENR-KD (+Dox) and DENR-Control (−Dox) HEK293T cells transiently transfected with reporter constructs as described in (**a**). The bar graphs show the calculated TRI mean values (n = 6). Immunoblots show levels of DENR and the β-actin loading control. (**g**) C33A cells with stable SBDS knockdown (shSBDS) or control cells (shCTRL) transiently transfected with reporter constructs as described in (**a**). The bar graphs show the calculated TRI mean values (n = 6). Immunoblots show levels of SBDS and the β-actin loading control. Statistical differences were analyzed by Student’s t-tests. Error bars represent ± SD, *P < 0.05, **P < 0. 01, ***P < 0. 001. Full scans of the immunoblots are presented in Supplementary Fig. S3.

The differential translation of the C/EBPβ-mRNA into LAP and LIP protein isoforms is under control of the mTORC1/4E-BP/eIF4E pathway^11^,^12^. To examine the performance of the reporter in response to mTORC1 hyperactivation we separately transfected the pcDNA-Firefly^Re-ini^/Renilla^Ini^ and pcDNA-Firefly^Ref^/Renilla^Ref^ reporter plasmids in mouse embryonic fibroblasts (MEFs) that are deficient in the mTORC1-inhibitory protein TSC1 (TSC1-KO)^26^ or in wt MEFs and calculated the TRI. Compared to the wt MEFs the TRI was increased in the TSC1-KO MEFs, revealing enhanced translation re-initiation, which also resulted in enhanced expression of LIP (Fig. 2a). A significant part of the mTORC1-dependent LIP regulation is mediated through the inhibitory phosphorylation of 4E-binding proteins (4E-BPs) by mTORC1, resulting in release of the eukaryotic translation initiation factor 4E (eIF-4E)^12^. In accordance, we found higher TRI values and increased LIP expression in 4E-BP1/2-double knockout (4E-BP1/2-DKO) MEFs comparing to wt MEFs (Fig. 2b). mTORC1 signaling can be pharmacologically inhibited by the allosteric inhibitor rapamycin or the catalytic inhibitor PP242. To examine the performance of the reporter under pharmacological conditions we generated HEK293T based cell lines with stable integrated pcDNA-Firefly^Re-ini^/Renilla^Ini^ or pcDNA-Firefly^Ref^/Renilla^Ref^ reporters. Treatment of the reporter cell lines with 200 nM rapamycin or 10 nM PP242 decreased LIP expression and lowered TRI values, revealing suppression of re-initiation under inhibited mTORC1 conditions (Fig. 2c). The mTORC1 inhibition was monitored by reduced phosphorylation of 4E-BP1 and S6 kinase (S6K). Finally, overexpression of eIF-4E that increases LIP expression accordingly increased TRI values (Fig. 2d). Therefore, the reporter system reliably reflects differential translation of the C/EBPβ-mRNA under changing mTORC1 signaling.

The eIF2α-kinases suppress post-uORF translation re-initiation through inhibitory phosphorylation of eIF2α in response to various stresses^13^,^14^, which results in reduced expression of LIP^11^. Augmenting eIF2 activity by treatment with the eIF2α-kinase inhibitor C16, which reduces eIF2α-phosphorylation, resulted in elevated translation re-initiation measured as higher TRI values and in an increase in LIP expression (Fig. 2e). Similarly, activation of eIF2α by overexpression of the dominant negative mutant of the eIF2α-kinase PKR (PKRΔ6) or the non-phosphorylatable eIF2αS51A mutant but not the phosphorylation mimicking eIF2αS51D mutant resulted higher TRI values (Fig. 2d). Thus, the alterations in eIF2α-kinase signaling can be measured using the TRI reporter system.

In addition to the canonical translation initiation pathways we examined the effects of two proteins that were shown to determine the efficiency of translation re-initiation by different mechanisms. The density-regulated protein (DENR) has been identified as a key regulator of eukaryotic re-initiation downstream of uORFs, and knockdown of DENR impairs the post-uORF re-initiation of translation^20^. To examine the effect of DENR expression we generated HEK293T cells with a doxycycline-inducible DENR-shRNA expression vector. Induction of DENR-KD (+Dox) resulted in lower TRI comparing to control cells (Fig. 2f), confirming the crucial role of DENR in uORF-dependent translation re-initiation. Recently, we have shown that deficiency in the Shwachman-Bodian-Diamond Syndrome (SBDS) protein impairs translation re-initiation into the C/EBPβ-LIP isoform^21^. In accordance, we measured lower TRI values in cells with shSBDS mediated knockdown of SBDS (Fig. 2g).

Taken together, the TRI reporter system reliably detects changes in the ratio of initiation versus re-initiation by known translationally active drugs or alterations in key translation initiation factors and their regulatory kinases or non-canonical regulators of re-initiation.

### Assay development and execution of a high-throughput screening (HTS)

To establish a reporter cell line suitable for high-throughput screening (HTS) we generated HEK293T cells with stably integrated pcDNA-Firefly^Re-ini^/Renilla^Ini^ reporter plasmid. Single clones were tested for renilla and firefly expression and the highly expressing cell clones were in addition tested for stability of expression over repeated cell passages. Cell line clones that met these criteria were selected for further experiments. Similarly, a cell line with stably integrated pcDNA-Firefly^Ref^/Renilla^Ref^ plasmid was generated. To determine an optimal assay window using reference compounds we performed kinetic experiments with the mTORC1 inhibitors rapamycin and PP242 over 24 hours. Both drugs showed the strongest decrease in TRI value at 8 hours, which was chosen as a suitable time point to perform the HTS (Supplementary Fig. S2a).

**Figure 3:**
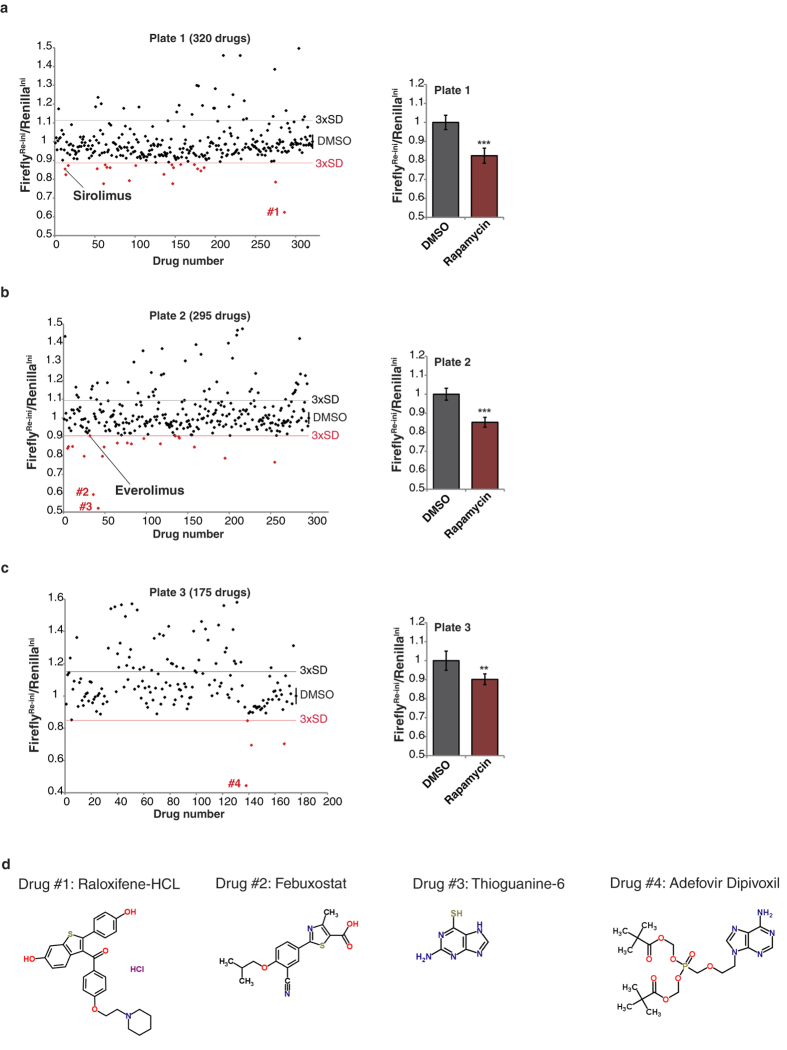
High throughput screening of a drug library. (**a**–**c**) Scatter plots for three different plates with measured translation re-initiation/initiation ratio (Firefly^Re-ini^/Renilla^Ini^) of 780 FDA approved drugs. Each plate contained 6 wells treated with DMSO as a solvent and rapamycin as a positive control (bar graph at the right, error bars represent SD). The threshold of three times the standard deviation from the DMSO mean value (3 × SD) is indicated. Drugs that decreased the translation re-initiation/initiation ratio more than 3xSD are indicated in red. Drugs indicated by numbers (#1, 2, 3 and 4) were selected for further evaluation. The arrows point to Sirolimus in plate 1 and Everolimus in plate 2. (**d**) Names and structural formulas of the drug #1–4 (retrieved from the ChemSpider database, www.chemspider.com). Statistical differences were analyzed by Student’s t-tests. Error bars represent ± SD (n = 6), **P < 0.01, ***P < 0.001.

We screened a library consisting of 780 FDA approved drugs (ENZO #BML-2841) using the Firefly^Re-ini^/Renilla^Ini^ HEK293T reporter cell line. 1.5 × 10^4^ cells per well (50 μl volume) were seeded in a 384-well format and the Firefly^Re-ini^/Renilla^Ini^ system was exposed to 10 μM of a single drug per well for 8 h (three assay plates in total). Each assay plate contained 6 wells with 0.5% v/v DMSO added as reference (Fig. 3a,b and c; bar graph at the right). Rapamycin was included as positive control on each plate (n = 6) and induced a significant decrease in re-initiation/initiation ratio (Firefly^Re-ini^/Renilla^Ini^) for each plate (plate 1, 0.82 ± 0.04; plate 2, 0.85 ± 0.025; plate 3, 0.9 ± 0.028) (Fig. 3a,b and c; bar graph at the right). 45 drugs were found to decrease the translation re-initiation/initiation ratio (Firefly^Re-ini^/Renilla^Ini^) below the threshold of three times the standard deviation from the mean value of DMSO treated cells (hit rate 5.7%) (Fig. 3a,b and c). The ENZO library contains three mTORC1 inhibitors that are derived from rapamycin (the rapalogs everolimus, sirolimus and temsirolimus) and two of them, sirolimus and everolimus, decreased re-initiation/initiation ratio (Fig. 3a and b). The temsirolimus signal was neglected because of aberrantly low luciferase signals (technical failure).

### Validation of the drugs that decrease translation re-initiation/initiation ratio

**Figure 4:**
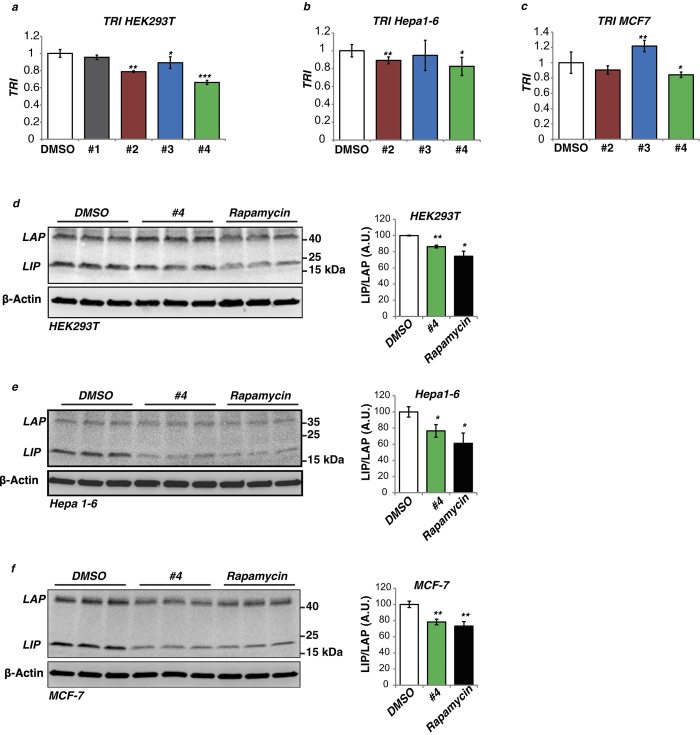
Validation of the drugs that decrease translation re-initiation/initiation ratio. (**a**) TRI mean values of HEK293T cells transiently transfected with pcDNA3-Firefly^Re-ini^/Renilla^Ini^ and pcDNA3-Firefly^Ref^/Renilla^Ref^ reporter plasmids and treated with drugs #1, 2, 3 and 4 (n = 4). (**b**,**c**) TRI mean values of Hepa1-6 or MCF7 cells transiently transfected with the constructs described in (**a**) and treated with drugs #2, 3 and 4 (n = 4). (**d**) Treatment with drug #4 (10 μM for 8 h) decreases C/EBPβ-LIP/LAP ratio in HEK293T cells as analyzed by immunoblotting. β-actin was used as a loading control. Bar chart at the right shows quantification of the relative changes in LIP/LAP-isoform ratio by drug #4 and rapamycin compared to DMSO solvent (n = 3). (**e**,**f**) Same experiment as described in (**d**) with Hepa1-6 or MCF7 cells, respectively (n = 3). C/EBPβ-LIP/LAP isoform ratios were quantified by chemiluminescence and digital imaging. Statistical differences were analyzed by Student’s t-tests. Error bars represent ± SD, *P < 0.05, **P < 0.01, ***P < 0. 001. Full scans of the immunoblots are presented in Supplementary Fig. S4.

Four drugs with the strongest effect on decreasing re-initiation/initiation ratio were chosen for further validation using counter assays (Fig. 3a–c, numbered values #1–4; Fig. 3d, drug names and structural formulas). Treatment of the pcDNA-Firefly^Ref^/Renilla^Ref^ or pcDNA3-Firefly^Re-ini^/Renilla^Ini^ containing HEK293T cell line using 10 μM of each drug for 8 h in a 96-well format revealed for drug #3 and #4 enhancement of the renilla reference signal compared to DMSO control (Supplementary Fig. S2b and c). The calculated TRIs (see formula Fig. 1b) revealed that similar to the result of the HTS screen drug #4 showed the strongest effect compared to drug #2 and #3, however, we could not confirm an effect of drug #1 on the TRI (Fig. 4a). Only drug #4 (Adefovir Dipivoxil) significantly lowered TRI values in two additional different cell lines, the mouse hepatoma cell line Hepa1-6 and the human breast cancer cell line MCF-7 (Fig. 4b and c). Drugs #2 did significantly lower the TRI in Hepa1-6 but not in MCF7 cells, and drug #3 did not alter the TRI in Hepa1-6 and even increased the TRI in MCF7 cells (Fig. 4b and c). These two drugs were excluded from further examination. To examine dose-dependency of treatment the TRI was measured for serially diluted concentrations between 1 nM and 100 μM for drugs #4 compared to drug #1 (Supplementary Fig. S2d). Drug #4 showed a dose-response with a half maximal effective concentration (EC_50_) of 1 μM, while drug #1 showed a much higher EC_50_ of 70.4 μM. Cytotoxicity over 8 hours treatment was examined using fluorescent cell viability assay (CellTiter-Fluor, Promega) and revealed for both drugs #1 and #4 only serious cytotoxicity at 100 μΜ concentrations (Supplementary Fig. S2e). In addition, treatment with drug #4 lowered endogenous LIP/LAP ratio comparable to treatment with rapamycin in HEK293T, Hepa 1–6 and MCF-7 cells (Fig. 4d,e and f). Taken together, the counter assays revealed that drug #4 is best in affect re-initiation measured as reduced TRI and lower LIP levels in cells that originate from three different tissues.

**Figure 5:**
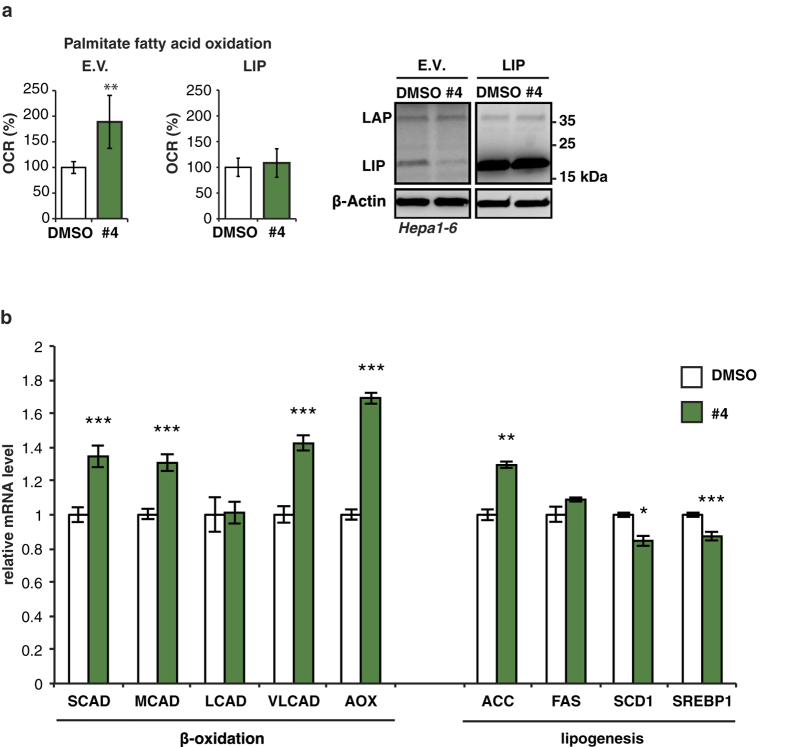
Drug #4 augments fatty acid β-oxidation. (**a**) Oxygen consumption rate (OCR) with palmitate as exogenous energy source for fatty acid β-oxidation in Hepa1-6 cells expressing either empty pcDNA3-vector control (E.V., left bar graph) or overexpressing LIP (right bar graph) and treated (over night) with drug #4 or DMSO solvent (n = 5). (**b**) Relative mRNA expression levels (qRT-PCR) of the β-oxidation genes SCAD, MCAD, LCAD, VLCAD, AOX, and lipogenesis genes ACC, FAS, SCD1 SREBP1c in HEPA1-6 cells treated with drug #4 or solvent DMSO (n = 3). Statistical differences were analyzed by Student’s t-tests. Error bars represent ± SD, *P < 0.05, **P < 0.01, ***P < 0. 001. Full scans of the immunoblots are presented in Supplementary Fig. S5.

We have recently shown that suppression of LIP expression results in a metabolic shift towards more fatty acid β-oxidation (FAO) in cell culture experiments and in a mouse model that is deficient in LIP expression (C/EBPβ^ΔuORF^ mice)^12^. To examine an eventual similar effect of drug #4 that also lowers LIP levels we measured FAO of exogenously added palmitate-BSA in Hepa1-6 cells using the Seahorse XF extracellular flux analyzer. Treatment with drug #4 resulted in an enhanced FAO-related oxygen consumption rate (OCR) that can be reverted by ectopic expression of LIP (Fig. 5a). The enhanced FAO phenotype of the C/EBPβ^ΔuORF^ mice is associated with upregulation of genes involved in FAO and unchanged or downregulated expression of lipogenesis genes in the liver^12^. Treatment of Hepa1-6 cells with drug #4 resulted in the upregulation of the FAO-related genes short-chain (SCAD), medium-chain (MCAD) and very long-chain (VLCAD) acyl-CoA dehydrogenases as well as acyl coenzyme A oxidase (AOX), but not of the long-chain acyl-CoA dehydrogenase (LCAD) gene (upregulated in C/EBPβ^ΔuORF^ mice) (Fig. 5b). In addition, the lipogenesis-related genes stearoyl-coenzyme A desaturase 1 (SCD1) and sterol regulatory element-binding protein 1c (SREBP1c) were modestly downregulated, fatty acid synthesis (FAS) was unchanged (downregulated in C/EBPβ^ΔuORF^ mice) and acetyl-CoA carboxylase (ACC) upregulated (unchanged in C/EBPβ^ΔuORF^ mice). Overall the effects of drug#4 on the examined genes show characteristics of enhanced FAO with a similar but not identical pattern compared to the C/EBPβ^ΔuORF^ mouse liver.

Taken together, our experiments show that the TRI reporter system can be successfully applied in HTS campaigns to identify compounds that alter the ratio between translation initiation and re-initiation depending on the C/EBPβ-uORF, which potentially display CR-mimetic properties.

## Discussion

We developed a cellular reporter system as a surrogate for measuring the ratio between translation initiation and uORF-dependent re-initiation as it is used as a mechanism for differential translation of the C/EBPβ-mRNA into the protein isoforms LAP and LIP. We have used reporter systems in the past based on C/EBPα-uORF regulation, however, these systems are not suitable for HTS^27^,^28^. Our main motivation to generate a C/EBPβ-based system came from our recent studies where we showed that experimental prevention of uORF-dependent translation re-initiation and the resulting decrease in LIP expression (decrease in LIP/LAP ratio) leads to CR-like metabolic improvements^12^,^17^,^29^. The uORF-dependent mechanism of LIP regulation seems reminiscent to the uORF-dependent induction of the nutrient-responsive yeast transcription factor Gcn4 in response to amino acid starvation and glucose limitation (dietary restriction). Gcn4 induction in yeast is required for full replicative lifespan extension in response to dietary restriction or depletion of 60 S ribosomes by deletion of ribosomal protein genes or treatment with the drug diazaborine^30^. Ribosomal re-initiation and expression of LIP is controlled by the mTORC1 pathway, linking the expression of this transcription factor to a crucial signaling network that determines metabolic health. Several studies have reported that LIP levels are reduced under conditions that promote metabolic health like CR whereas increased LIP levels are associated with detrimental health conditions like high fat diets, aging and cancer^12^,^18^,^31^,^32^,^33^. Therefore, we reasoned that suppression of LIP function by translational downregulation might have therapeutic value since it mimics effects of CR. The specific uORF-dependent translation into LIP can be targeted since we know that pharmacological inhibition of mTORC1 or genetic alterations in certain translation initiation factors results in suppression of LIP translation. Reducing LIP levels through the well-known inhibition of mTORC1 by rapamycin and derivatives (rapalogs) is not optimal since the response varies widely between cell types^34^,^35^ and treatment comes with a diversity of serious side effects^22^,^23^,^24^,^25^,^36^,^37^. Since other mechanisms that may impact on LIP translation are not well understood and partially obscure we set out to develop a phenotypic screening system for the identification of small molecule compounds that reduce translation re-initiation that is under control of the C/EBPβ-uORF, in an unbiased way. To validate the functionality of the screening system we chose several established genetic alterations and pharmacological treatments that alter LIP translation via different pathways to show that the cellular Firefly^Re-ini^/Renilla^Ini^ luciferase reporter reliably emulates the two different translation events from the C/EBPβ-mRNA; firefly expression through re-initiation and renilla expression through initiation.

The screening of novel targets against known drugs offers the potential for drug repurposing with reduced cost and time scale for evaluation against alternative diseases^38^. For an initial screen we therefore chose a small molecule library of 780 FDA approved drugs (ENZO #BML-2841) using a HEK293T-based clonal cell line with stably integrated pcDNA3-Firefly^Re-ini^/Renilla^Ini^ reporter plasmid. Hits were classified as those compounds that resulted in a reduced Firefly^Re-ini^/Renilla^Ini^ signal compared to the DMSO control with values larger than three times standard deviation (3xSD), in total 45 drugs (5.7% hit rate). A selection of four drugs that showed the strongest reduction in Firefly^Re-ini^/Renilla^Ini^ signal was subjected to counter assays. Drug #4 was the sole drug to pass all tests and reducing the TRI and LIP levels in three different cell lines in comparison with rapamycin that showed similar effects. It is off course possible that additional drugs that also fulfill these criteria are among the other 41 identified but not yet re-tested drugs.

A prominent effect of reducing LIP levels at both cellular and organismal levels is an enhanced fatty acid oxidation, which beneficially influences metabolic health^12^. Drug #4 induced a similar increase in fatty acid oxidation in a liver cell line, which could be reversed by overexpression of LIP. Taken together, we identified drug #4 from a collection of 780 FDA approved drugs, which reduces C/EBPβ-uORF-dependent translation re-initiation and enhanced generation of the protein isoform LIP with concomitant associated enhancement of fatty acid oxidation. Drug treatment experiments in mice will have to clarify whether candidate drugs alter systemic metabolism comparable to the effects found in the C/EBPβ^ΔuORF^ mice^12^. It is beyond the scope of this manuscript to identify the underlying molecular target(s) or mechanism of action of drug #4 (or one of the other candidates), or to address the potential for drug repurposing. In addition, all 45 hits should be subject of further validation and characterization. Nonetheless, the data do show that the system can be used for HTS campaigns to identify small molecule compounds that reduce LIP levels and potentially have CR-mimetic properties. This is important, since the pharmacological options to utilize the beneficial effects of CR-type metabolic alterations and its related cancer reducing properties are limited.

The system we present here belongs to the type of phenotypic drug discovery (forward pharmacology) where compounds are identified that cause a desirable change in phenotype, here reduction of C/EBPβ-uORF-dependent translation re-initiation. The system, however, is not purely phenotypic and target-agnostic (without having prior knowledge of their biological activity or mode of action against a specific molecular target or targets) and is better described as a translation re-initiation and initiation mechanism-informed phenotypic screen^39^,^40^. Phenotypic screens potentially can identify compounds that modify a (disease) phenotype by acting on a yet undiscovered target or targets. The successive identification of the target(s) using for example techniques as chemical proteomics^41^ could give novel insights into translation control mechanisms and involved upstream signaling pathways and might thereby also broaden our knowledge of potential players in metabolic diseases, aging and cancer.

## Methods

### DNA constructs

For generation of pcDNA-Firefly^Re-ini^/Renilla^Ini^, we first cloned the rat C/EBPβ-5′-leader sequence until the LAP initiation codon in frame with the renilla sequence (from pGL4) in pcDNA3, and separately cloned C/EBPβ-sequences spanning the 5-leader and sequences until the LIP initiation codon with a +1 frame shift (7 nt upstream of the AUG) with the firefly sequences (from pGL3) in pcDNA3. A fragment of the pcDNA3-LAP-Renilla between the BglII restriction site and spanning the renilla sequences was amplified by PCR using the following primers, *forward*: 5′-CGG AAA TGT TGA ATA CTC ATA CTC-′3 and *reverse*: 5′-GCT CAG ATC TCC TCA GAA GCC ATA GAG C-′3. The amplified fragment was sequenced and cloned in the pcDNA3-LIP-Firefly using the BglII site to obtain the final pcDNA-Firefly^Re-ini^/Renilla^Ini^. For the reference construct pcDNA-Firefly^Ref^/Renilla^Ref^, a similar cloning strategy was employed using solely renilla and firefly coding regions (from pGL4 and pGL3 respectively). Details of the cloning strategy will be provided upon request. eIF4E, eIF2α-S51D, eIF2α-S51A and PKRΔ6 pcDNA3-based expression vectors are described in ref. 11. The human DENR shRNA expression vector was generated by annealing the oligonucleotides shDENR *forward*: ‘5-CCG GCA AGA GTA TGT GGC CTT GCA ACT CGA GTT GCA AGG CCA CAT ACT CTT GTT TTT T-3’ and shDENR *reverse*: ‘5-AAT TAA AAA ACA AGA GTA TGT GGC CTT GCA ACT CGA GTT GCA AGG CCA CAT ACT CTT G-3’ and ligating them into the Tet-pLKO-puro vector (Addgene plasmid #21915, described in ref. 42).

### Cell culture

HEK293T and Hepa1-6 cells were maintained in DMEM and MCF7 cells in RPMI (Gibco) supplemented with 10% fetal calf serum (FCS) and 1% Penicillin/Streptomycin at 37 °C with 5% CO2. TSC1-KO and 4E-BP1/2-DKO MEFs were previously described^43^,^44^. SBDS-KD C33A cells were used before^21^. Hepa1-6 cells ectopically expressing C/EBPβ-LIP from a pcDNA3 expression vector were described at ref. 12. Cell number and viability was determined using the TC20^TM^ automated cell counter (Bio-Rad) following the manufacturers instruction. For generation of cells stably expressing the reporter constructs, pcDNA-Firefly^Re-ini^/Renilla^Ini^ or pcDNA-Firefly^Ref^/Renilla^Ref^ were transfected into HEK293T cells using FUGENE (Roche). Cell clones were selected by addition of G418 to the medium (1 mg/ml). For knockdown of DENR in HEK293T cells, the cells were transduced with the inducible lentiviral Tet-pLKO-puro-shDENR followed by selection of the transduced cells with puromycin (1.5 μg/ml). Then these cells were treated with doxycycline (100 ng/ml) for 36 h. Rapamycin (R-5000, LC Laboratories), PP242 (#P0073, Sigma) and eIF-2α-kinase PKR inhibitor (C16) (#I9785, Sigma) were used as chemical inhibitors.

### Luciferase assay

For the Luciferase assay, 25000 cells per well were seeded in 96-well plates. After 24 h, cells were cotransfected with the plasmids as indicated using FUGENE HD (Promega), or stably transfected cells were used. The next day, luciferase activity was measured in cells treated with 8 h of drug treatment or DMSO solvent control by Dual-Glo Luciferase Assay System (#2920, Promega) following the manufacturer’s protocol using a GloMax-Multi Detection System (Promega).

### Viability Assay

For the viability assay, 40000 HEK293T cells were seeded in 96-well plates. After 24 h, cells were treated with the different drugs for 8 h and cell viability was measured by Cell-Titer Flour cell viability assay system (#G6081, Promega) following the manufacturer’s protocol using a GloMax-Multi Detection System (Promega).

### Immunoblotting

For protein extraction, the cells were washed twice with ice-cold 1 × PBS and lysed in 50 mM Tris pH 7.5, 150 mM NaCl, 1 mM EDTA, 1% Triton X-100, supplemented with protease and phosphatase inhibitors followed by sonication. Equal amounts of total protein were separated by SDS–PAGE (#456-1094, BIO-RAD), transferred to a PVDF membrane using Trans-Blot Turbo System (#170-4273, BIO-RAD) following the manufacturer’s protocol. The following antibodies were used: C/EBPβ (C19) and SBDS (S-15) from Santa Cruz Biotechnology; phospho-p70S6K (Thr389) (108D2), p70S6K (#9202), Hamartin/TSC1 (D43E2) (#6935), phospho-4E-BP1 (Thr37/46) (#9459), 4E-BP1 (#9452), 4E-BP2 (#2845), eIF4E (#9742), Phospho-eIF2α (Ser51) (#9721), eIF2α (#9722) and PKR (#3072) from Cell Signaling; DENR (#10656-1-AP) from Proteintech^TM^ and β-actin (clone C4) (#691001) from MP Biomedicals. HRP-conjugated secondary antibodies were purchased from Amersham Life Technologies. The bands were visualized by chemiluminescence (ECL, Amersham Life Technologies) using ImageQuant LAS 4000 mini imaging machine (GE Healthcare Bioscience AB) and the supplied software was used for the quantification of the bands.

### Library screening

HEK293T cells stably expressing Firefly^Re-ini^/Renilla^Ini^ vector were seeded at a density of 1.5 × 10^4^ in 30 μl media per well of a 384-well plate (Greiner CELLSTAR^®^ 384 well plates, white) and grown overnight. Next day, the cells were treated for eight hours with 780 FDA approved drugs library (#BML-2843-0100, Enzo Lifescience) (final concentration 10 μM), 0.5% v/v DMSO and rapamycin (final concentration 200 nM) using an Echo^®^ 555 Liquid Handler (Labcyte) transferring up to 150 nl to each well. After lysis of the cells, luciferase activity was measured.

### Measurements of cellular oxygen consumption rate and fatty acid oxidation

Oxygen consumption rate and fatty acid oxidation were determined using a Seahorse XF96 Extracellular Flux analyzer (Seahorse Bioscience). For fatty acid oxidation assay, 2 × 10^4^ Hepa 1–6 cells per well were seeded into a 96-well XF cell culture microplate 24 h prior to the assay and cultured in the presence of 0.5 mM carnitine. Sixteen hours before the assay, cells were treated with the drugs. One hour before the assay, the cells were washed twice with FAO assay buffer (Seahorse Bioscience), and 15 min before the assay, the fatty acid oxidation substrate palmitate-BSA or a BSA control (Seahorse Bioscience) was added and the oxygen consumption rate (OCR) with or without palmitate-BSA was measured.

#### Quantitative Real-Time PCR analysis

Total RNA was isolated using the RNeasy Kit (QIAGEN). For cDNA synthesis 1 μg RNA was reverse transcribed with the Transcriptor First Strand cDNA Synthesis Kit (Roche) using Oligo(d)T primers. qRT-PCR was performed using the LightCycler^®^ 480 SYBR Green I Master Mix (Roche). The following primer pairs were used: β-actin (normalization): 5′-aga ggg aaa tcg tgc gtg ac-3′ and 5′-caa tag tga tga cct ggc cgt-3′, SCAD: 5′-cct gca acc gag aag aaa tc-3′ and 5′-cct gtc ctg tcc ctt gtg tt-3′, MCAD: 5′-ggt ttg gct ttt gga caa tg-3′ and 5′-tga cgt gtc caa tct acc aca-3′, LCAD: 5′-gct gcc ctc ctc ccg atg tt-3′ and 5′-atg ttt ctc tgc gat gtt gat g-3′, VLCAD: 5′-ttg tca acg agc agt tcc tg-3′ and 5′-agc ctc aat gca cca gct at-3′, AOX: 5′-aag agt tca ttc tca aca gcc c-3′ and 5′-ctt gga cag act ctg agc tgc-3′, ACC: 5′-ggg act tca tga att tgc tga ttc tca gtt-3′ and 5′-gtc att acc atc ttc att acc tca atc tc-3′, FAS: 5′-aca cag caa ggt gct gga g-3′ and 5′-gtc cag gct gtg gtg act ct-3′, SCD1: 5′-ccg gag acc ctt aga tcg a-3′ and 5′-tag cct gta aaa gat ttc tgc aaa cc-3′, SREBP1c: 5′-aac gtc act tcc agc tag ac-3′ and 5′-cca cta agg tgc cta cag agc-3′.

## Additional Information

**How to cite this article**: Zaini, M. A. *et al*. A screening strategy for the discovery of drugs that reduce C/EBPβ-LIP translation with potential calorie restriction mimetic properties. *Sci. Rep.* **7**, 42603; doi: 10.1038/srep42603 (2017).

**Publisher's note:** Springer Nature remains neutral with regard to jurisdictional claims in published maps and institutional affiliations.

## Supplementary Material

Supplementary Figures

## References

[b1] Fontana L., Partridge L. & Longo V. D. Extending healthy life span–from yeast to humans. Science 328, 321–326, doi: 10.1126/science.1172539 (2010). 20395504 PMC3607354

[b2] Zoncu R., Efeyan A. & Sabatini D. M. mTOR: from growth signal integration to cancer, diabetes and ageing. Nat Rev Mol Cell Biol 12, 21–35, doi: 10.1038/nrm3025 (2011). 21157483 PMC3390257

[b3] Johnson S. C., Rabinovitch P. S. & Kaeberlein M. mTOR is a key modulator of ageing and age-related disease. Nature 493, 338–345, doi: 10.1038/nature11861 (2013). 23325216 PMC3687363

[b4] Kennedy B. K. & Lamming D. W. The Mechanistic Target of Rapamycin: The Grand ConducTOR of Metabolism and Aging. Cell Metab 23, 990–1003, doi: 10.1016/j.cmet.2016.05.009 (2016). 27304501 PMC4910876

[b5] Kapahi P. . Regulation of lifespan in Drosophila by modulation of genes in the TOR signaling pathway. Curr Biol 14, 885–890, doi: 10.1016/j.cub.2004.03.059 (2004). 15186745 PMC2754830

[b6] Selman C. . Ribosomal protein S6 kinase 1 signaling regulates mammalian life span. Science 326, 140–144, doi: 10.1126/science.1177221 (2009). 19797661 PMC4954603

[b7] Solon-Biet S. M. . The ratio of macronutrients, not caloric intake, dictates cardiometabolic health, aging, and longevity in ad libitum-fed mice. Cell Metab 19, 418–430, doi: 10.1016/j.cmet.2014.02.009 (2014). 24606899 PMC5087279

[b8] Wu J. J. . Increased mammalian lifespan and a segmental and tissue-specific slowing of aging after genetic reduction of mTOR expression. Cell Rep 4, 913–920, doi: 10.1016/j.celrep.2013.07.030 (2013). 23994476 PMC3784301

[b9] Guarente L. Calorie restriction and sirtuins revisited. Genes Dev 27, 2072–2085, doi: 10.1101/gad.227439.113 (2013). 24115767 PMC3850092

[b10] Shimobayashi M. & Hall M. N. Making new contacts: the mTOR network in metabolism and signalling crosstalk. Nat Rev Mol Cell Biol 15, 155–162, doi: 10.1038/nrm3757 (2014). 24556838

[b11] Calkhoven C. F., Muller C. & Leutz A. Translational control of C/EBPalpha and C/EBPbeta isoform expression. Genes Dev 14, 1920–1932 (2000). 10921906 PMC316813

[b12] Zidek L. M. . Deficiency in mTORC1-controlled C/EBPbeta-mRNA translation improves metabolic health in mice. EMBO Rep 16, 1022–1036, doi: 10.15252/embr.201439837 (2015). 26113365 PMC4552494

[b13] Aitken C. E. & Lorsch J. R. A mechanistic overview of translation initiation in eukaryotes. Nat Struct Mol Biol 19, 568–576, doi: 10.1038/nsmb.2303 (2012). 22664984

[b14] Sonenberg N. & Hinnebusch A. G. Regulation of translation initiation in eukaryotes: mechanisms and biological targets. Cell 136, 731–745, doi: 10.1016/j.cell.2009.01.042 (2009). 19239892 PMC3610329

[b15] Descombes P. & Schibler U. A liver-enriched transcriptional activator protein, LAP, and a transcriptional inhibitory protein, LIP, are translated from the same mRNA. Cell 67, 569–579 (1991). 1934061 10.1016/0092-8674(91)90531-3

[b16] Wethmar K. . C/EBPbetaDeltauORF mice–a genetic model for uORF-mediated translational control in mammals. Genes Dev 24, 15–20, doi: 10.1101/gad.557910 (2010). 20047998 PMC2802187

[b17] Albert V. & Hall M. N. Reduced C/EBPbeta-LIP translation improves metabolic health. EMBO Rep 16, 881–882, doi: 10.15252/embr.201540757 (2015). 26113367 PMC4552476

[b18] Begay V. . Deregulation of the endogenous C/EBPbeta LIP isoform predisposes to tumorigenesis. J Mol Med (Berl) 93, 39–49, doi: 10.1007/s00109-014-1215-5 (2015). 25401168

[b19] Barbosa C., Peixeiro I. & Romao L. Gene expression regulation by upstream open reading frames and human disease. PLoS Genet 9, e1003529, doi: 10.1371/journal.pgen.1003529 (2013). 23950723 PMC3738444

[b20] Schleich S. . DENR-MCT-1 promotes translation re-initiation downstream of uORFs to control tissue growth. Nature 512, 208–212, doi: 10.1038/nature13401 (2014). 25043021 PMC4134322

[b21] In K. . Shwachman-Bodian-Diamond syndrome (SBDS) protein deficiency impairs translation re-initiation from C/EBPalpha and C/EBPbeta mRNAs. Nucleic Acids Res 44, 4134–4146, doi: 10.1093/nar/gkw005 (2016). 26762974 PMC4872075

[b22] Stallone G., Infante B., Grandaliano G. & Gesualdo L. Management of side effects of sirolimus therapy. Transplantation 87, S23–26, doi: 10.1097/TP.0b013e3181a05b7a (2009). 19384183

[b23] Zaytseva Y. Y., Valentino J. D., Gulhati P. & Evers B. M. mTOR inhibitors in cancer therapy. Cancer Lett 319, 1–7, doi: 10.1016/j.canlet.2012.01.005 (2012). 22261336

[b24] Willemsen A. E. . mTOR inhibitor-induced interstitial lung disease in cancer patients: Comprehensive review and a practical management algorithm. Int J Cancer 138, 2312–2321, doi: 10.1002/ijc.29887 (2016). 26452336

[b25] Wander S. A., Hennessy B. T. & Slingerland J. M. Next-generation mTOR inhibitors in clinical oncology: how pathway complexity informs therapeutic strategy. J Clin Invest 121, 1231–1241, doi: 10.1172/JCI44145 (2011). 21490404 PMC3069769

[b26] Kwiatkowski D. J. . A mouse model of TSC1 reveals sex-dependent lethality from liver hemangiomas, and up-regulation of p70S6 kinase activity in Tsc1 null cells. Hum Mol Genet 11, 525–534 (2002). 11875047 10.1093/hmg/11.5.525

[b27] Wiesenthal V., Leutz A. & Calkhoven C. F. Analysis of translation initiation using a translation control reporter system. Nat Protoc 1, 1531–1537, doi: 10.1038/nprot.2006.274 (2006). 17406445

[b28] Wiesenthal V., Leutz A. & Calkhoven C. F. A translation control reporter system (TCRS) for the analysis of translationally controlled processes in the vertebrate cell. Nucleic Acids Res 34, e23, doi: 10.1093/nar/gnj029 (2006). 16473846 PMC1363784

[b29] Zahnow C. A., Younes P., Laucirica R. & Rosen J. M. Overexpression of C/EBPbeta-LIP, a naturally occurring, dominant-negative transcription factor, in human breast cancer. J Natl Cancer Inst 89, 1887–1891 (1997). 9414177 10.1093/jnci/89.24.1887

[b30] Steffen K. K. . Yeast life span extension by depletion of 60s ribosomal subunits is mediated by Gcn4. Cell 133, 292–302, doi: 10.1016/j.cell.2008.02.037 (2008). 18423200 PMC2749658

[b31] Karagiannides I. . Altered expression of C/EBP family members results in decreased adipogenesis with aging. Am J Physiol Regul Integr Comp Physiol 280, R1772–1780 (2001). 11353682 10.1152/ajpregu.2001.280.6.R1772

[b32] Timchenko L. T. . Age-specific CUGBP1-eIF2 complex increases translation of CCAAT/enhancer-binding protein beta in old liver. J Biol Chem 281, 32806–32819, doi: 10.1074/jbc.M605701200 (2006). 16931514

[b33] Esteves C. L. . Regulation of adipocyte 11beta-hydroxysteroid dehydrogenase type 1 (11beta-HSD1) by CCAAT/enhancer-binding protein (C/EBP) beta isoforms, LIP and LAP. PLoS One 7, e37953, doi: 10.1371/journal.pone.0037953 (2012). 22662254 PMC3360670

[b34] Thoreen C. C. . An ATP-competitive mammalian target of rapamycin inhibitor reveals rapamycin-resistant functions of mTORC1. J Biol Chem 284, 8023–8032, doi: 10.1074/jbc.M900301200 (2009). 19150980 PMC2658096

[b35] Thoreen C. C. & Sabatini D. M. Rapamycin inhibits mTORC1, but not completely. Autophagy 5, 725–726 (2009). 19395872 10.4161/auto.5.5.8504

[b36] Arriola Apelo S. I. & Lamming D. W. Rapamycin: An InhibiTOR of Aging Emerges From the Soil of Easter Island. J Gerontol A Biol Sci Med Sci 71, 841–849, doi: 10.1093/gerona/glw090 (2016). 27208895 PMC4906330

[b37] Lamming D. W., Ye L., Sabatini D. M. & Baur J. A. Rapalogs and mTOR inhibitors as anti-aging therapeutics. J Clin Invest 123, 980–989, doi: 10.1172/JCI64099 (2013). 23454761 PMC3582126

[b38] Ashburn T. T. & Thor K. B. Drug repositioning: identifying and developing new uses for existing drugs. Nat Rev Drug Discov 3, 673–683, doi: 10.1038/nrd1468 (2004). 15286734

[b39] Moffat J. G., Rudolph J. & Bailey D. Phenotypic screening in cancer drug discovery - past, present and future. Nat Rev Drug Discov 13, 588–602, doi: 10.1038/nrd4366 (2014). 25033736

[b40] Swinney D. C. & Anthony J. How were new medicines discovered? Nat Rev Drug Discov 10, 507–519, doi: 10.1038/nrd3480 (2011). 21701501

[b41] Rix U. & Superti-Furga G. Target profiling of small molecules by chemical proteomics. Nat Chem Biol 5, 616–624, doi: 10.1038/nchembio.216 (2009). 19690537

[b42] Wiederschain D. . Single-vector inducible lentiviral RNAi system for oncology target validation. Cell Cycle 8, 498–504, doi: 10.4161/cc.8.3.7701 (2009). 19177017

[b43] Le Bacquer O. . Elevated sensitivity to diet-induced obesity and insulin resistance in mice lacking 4E-BP1 and 4E-BP2. J Clin Invest 117, 387–396, doi: 10.1172/JCI29528 (2007). 17273556 PMC1783830

[b44] Zhang H. . Loss of Tsc1/Tsc2 activates mTOR and disrupts PI3K-Akt signaling through downregulation of PDGFR. J Clin Invest 112, 1223–1233, doi: 10.1172/JCI17222 (2003). 14561707 PMC213485

