# Supplementary material for: A screening strategy for the discovery of drugs that reduce C/EBPβ-LIP translation with potential calorie restriction mimetic properties
Source: Sci Rep. 2017 Feb 15;7:42603. doi: 10.1038/srep42603 (PMC5309760; doi:10.1038/srep42603)
Supplement: Supplementary Figures [file srep42603-s1.pdf]

Supplementary Figures

A screening strategy for the discovery of drugs that reduce C/EBPβ-LIP translation with potential calorie restriction mimetic properties

Mohamad A Zaini, Christine Müller, Tobias Ackermann, Jeanette Reinshagen, Gertrud Kortman, Ole Pless, Cornelis F Calkhoven

Figure S1

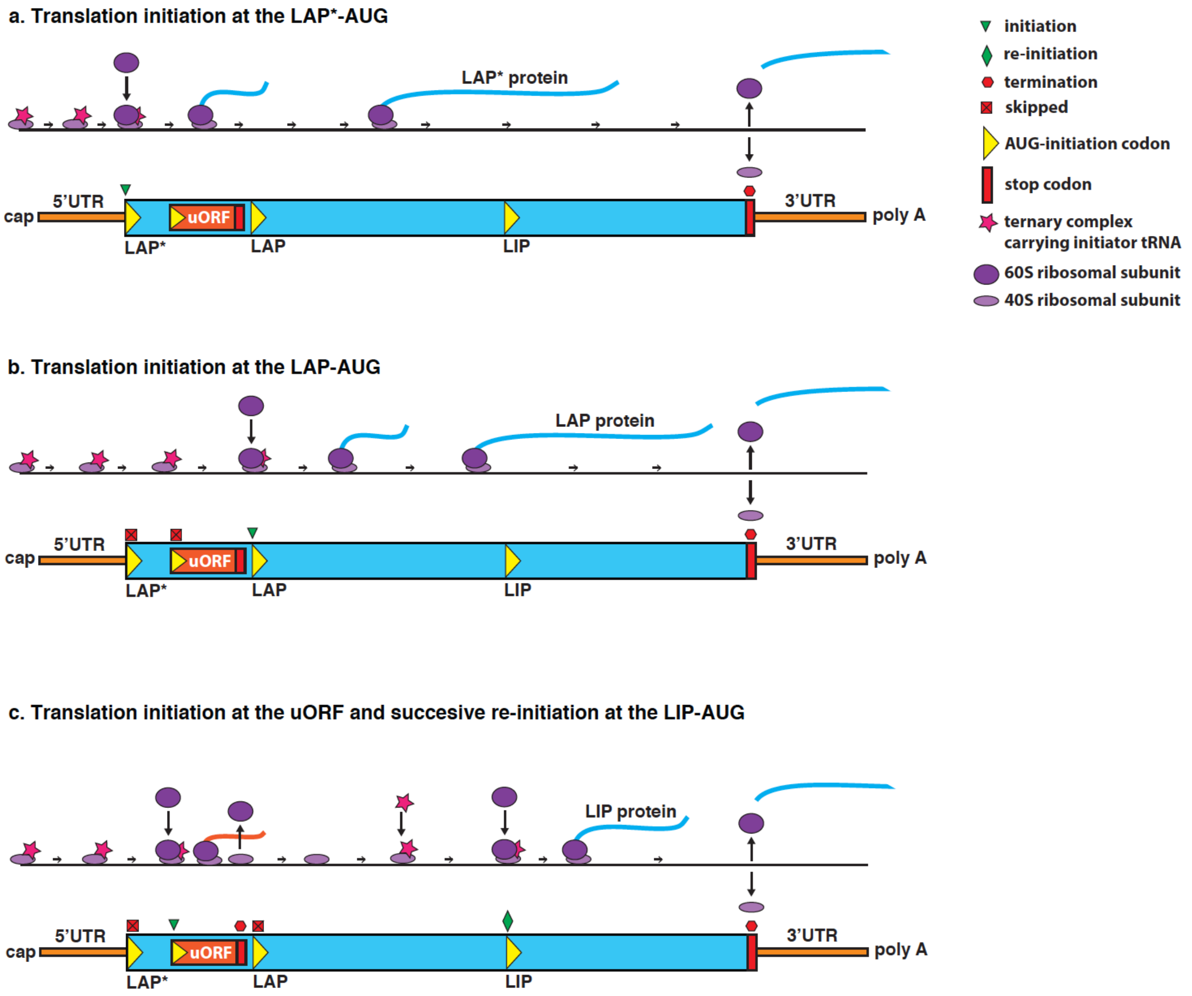

Figure S1: Translation initiation and re-initiation from the C/EBPβ mRNA. (**a**) Translation of C/EBPβ-LAP* by translation initiation. Ribosomes scan the mRNA from the 5'-cap to the LAP*-AUG to initiate translation. LAP* is often weakly expressed because it has no Kozak sequence. (**b**) Translation of C/EBPβ-LAP by translation initiation. Ribosomes scan the mRNA from the 5'-cap omitting the LAP*-AUG and uORF-AUG in order to initiate translation at the LAP-AUG which has a favorable Kozak sequence. (**c**) Translation of C/EBPβ-LIP by translation re-initiation. Ribosomes scan the mRNA from the 5'-cap omitting the LAP*-AUG to initiate the translation from the uORF-AUG. After producing a small uORF peptide, the translation is terminated and the post-termination ribosomes restart scanning the mRNA omitting the nearby (4nt downstream) LAP-AUG. During the re-scanning, the post-termination ribosomes are reloaded by a new initiator tRNA (Met-tRNAiMet) required for translation re-initiation at the LIP-AUG.

Figure S2

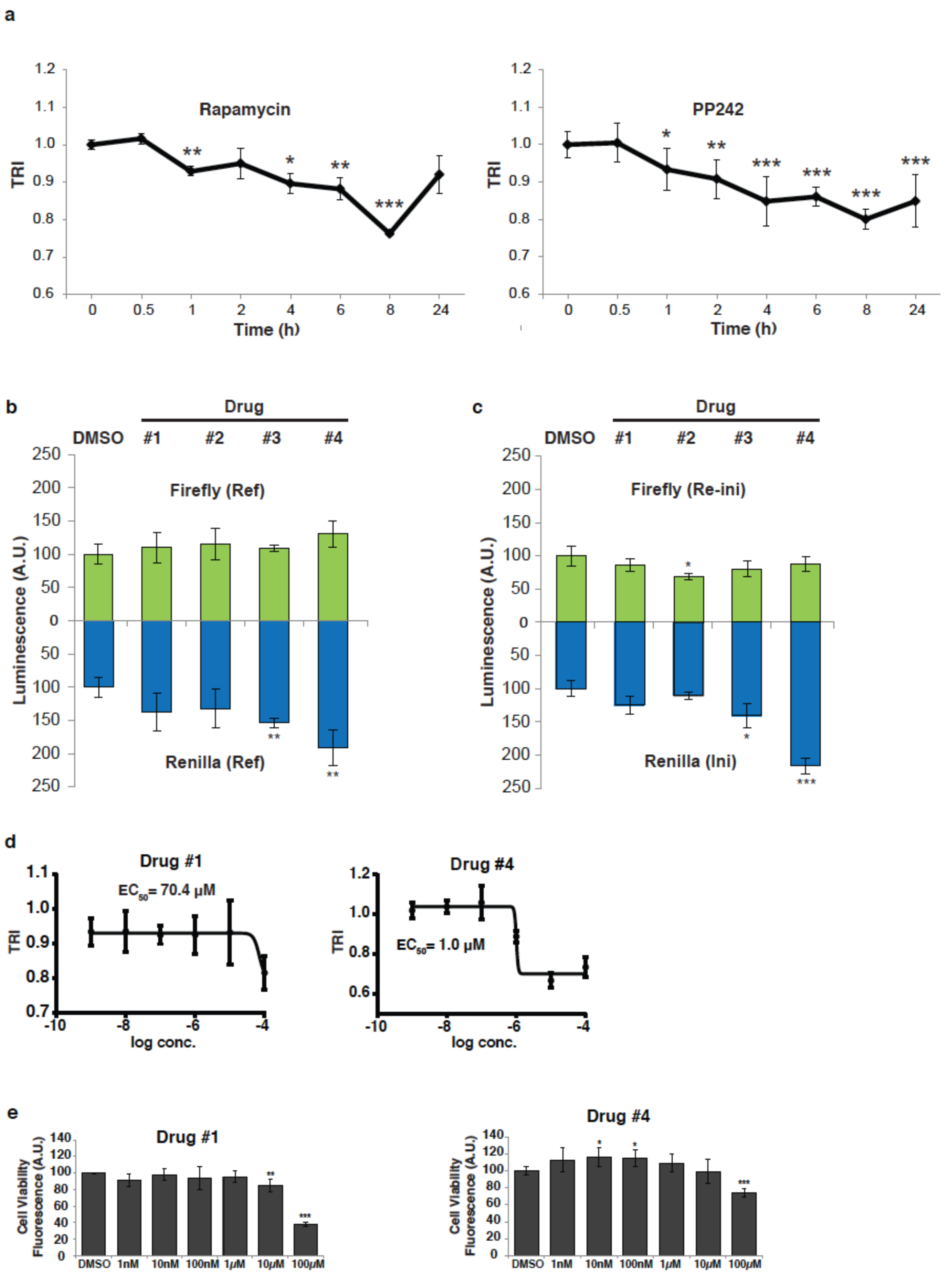

Figure S2: Various drug treatment assays. (**a**) Treatment kinetics of the mTORC1 inhibitors rapamycin (200 nM) or PP242 (10 nM) over 24 hours using pcDNA3-FireflyRe-ini/RenillaIni and pcDNA-FireflyRef/RenillaRef reporter HEK293T cells and calculated TRI (n=3). Both drugs showed the strongest decrease in TRI value at 8 hours, which was chosen as a suitable time point to perform the HTS. (**b**) Effects of drugs #1-4 (10 µM) on firefly or renilla luciferase expression in pcDNA-FireflyRef/RenillaRef control reporter HEK293T cells (n=4). (**c**) Effects of drugs #1-4 (10 µM) on firefly or renilla luciferase expression in pcDNA3-FireflyRe-ini/RenillaIni reporter HEK293T cells. (**d**) Determination of EC50 values for drugs #1 and #4 (n=5). (**e**) Determination of cell toxicity (CellTiter-Fluor, Promega) for drugs #1 and #4 (n=5). Statistical differences were analyzed by Student's t-tests. Error bars represent ±SD, *P < 0.05, **P < 0.01, ***P < 0. 001.

Figure S3

Full scans Figure 2a

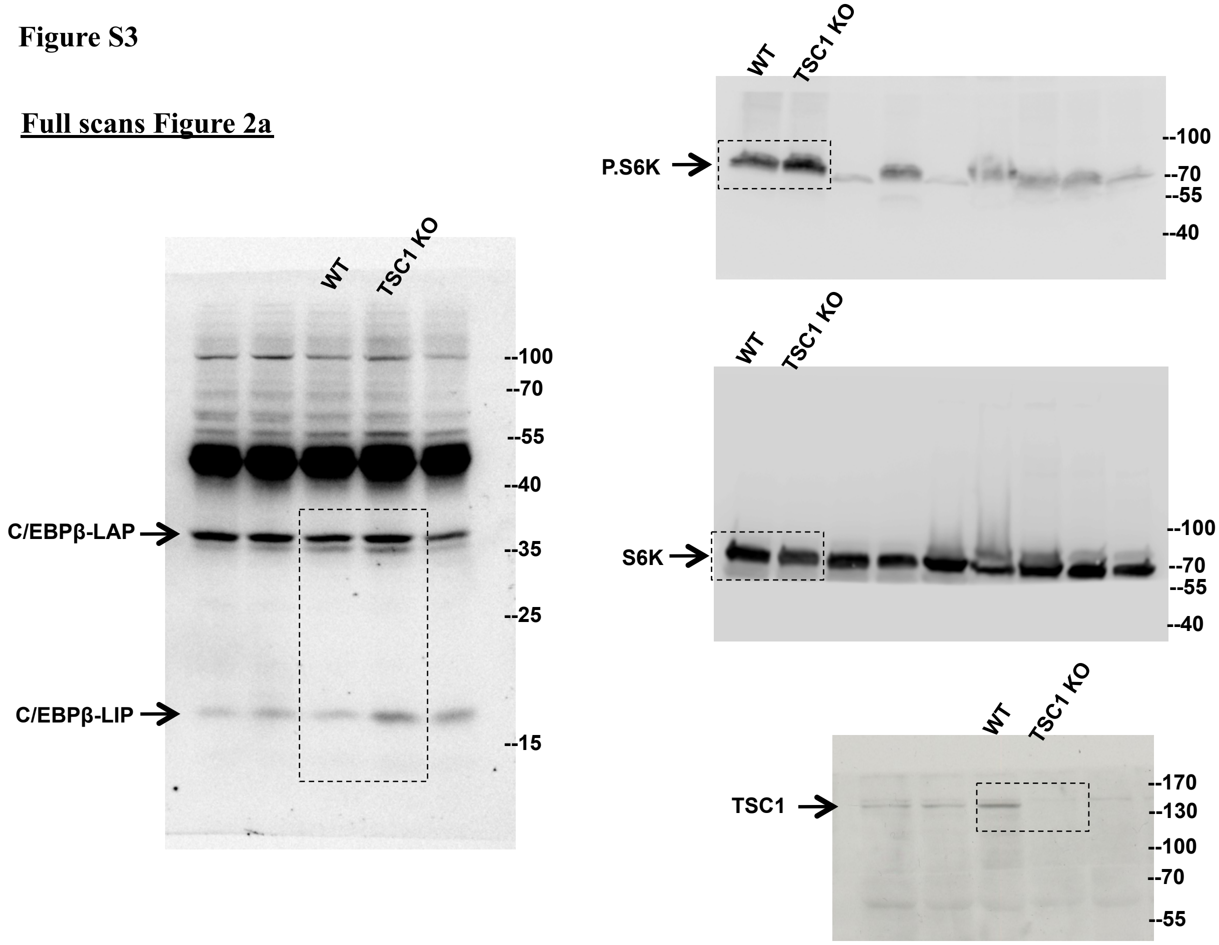

Full scans Figure 2b

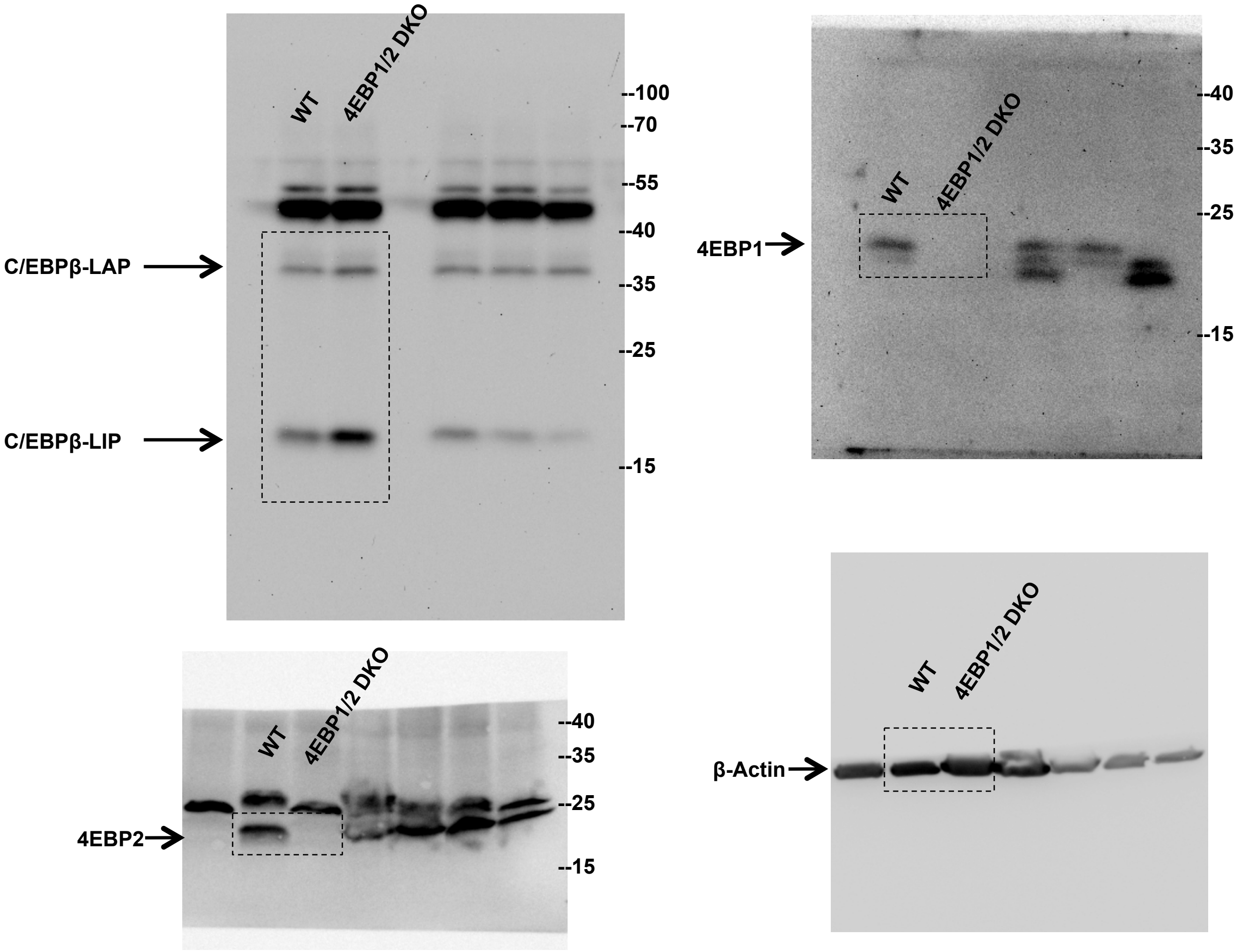

Figure S3

Full scans Figure 2c

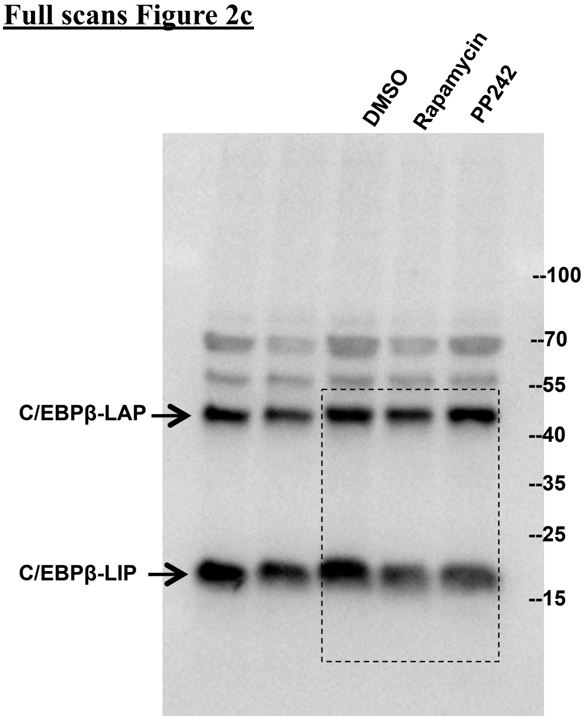

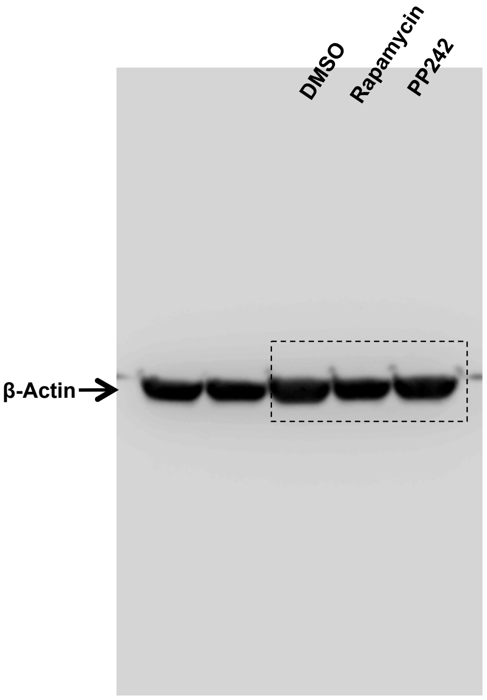

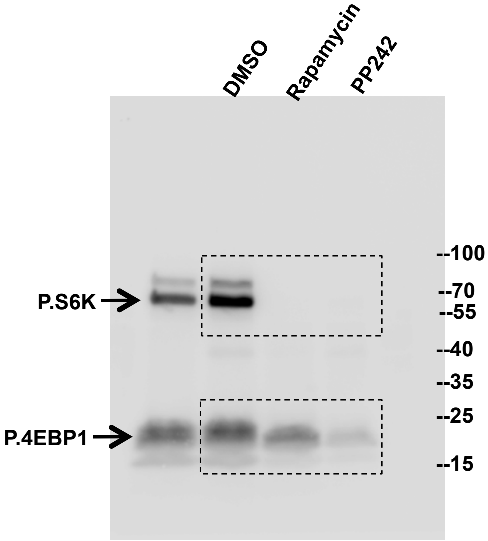

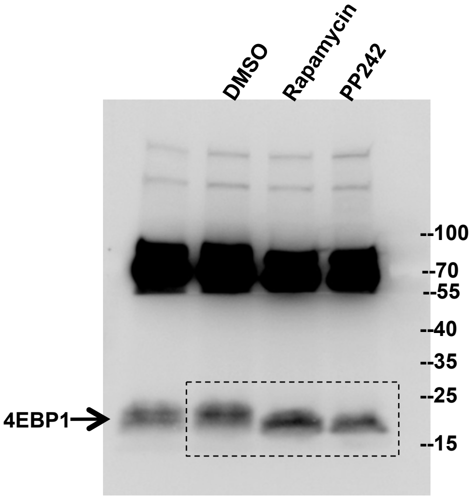

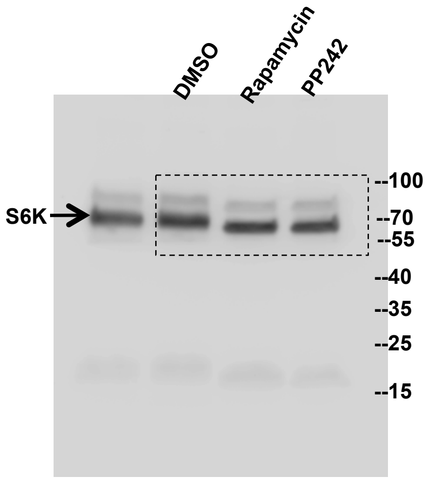

Figure S3

Full scans Figure 2d

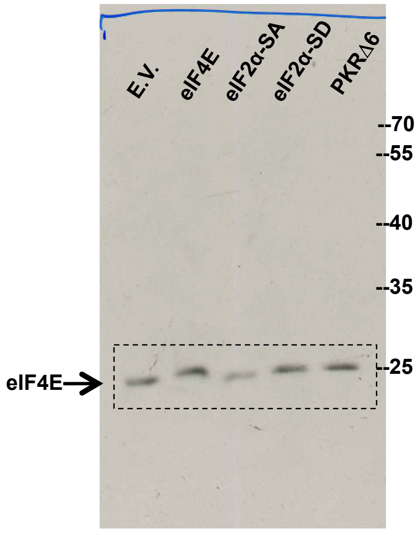

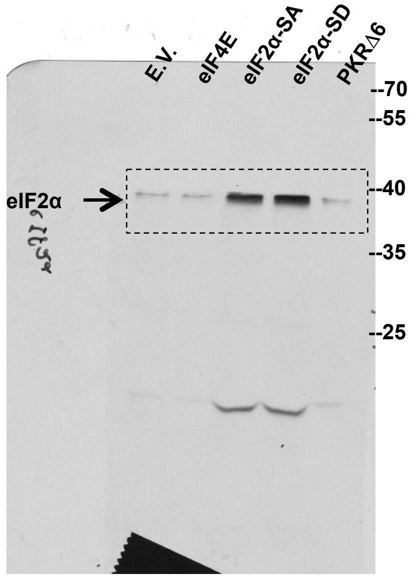

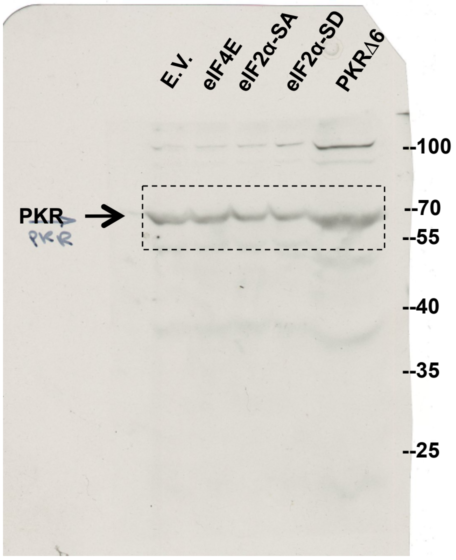

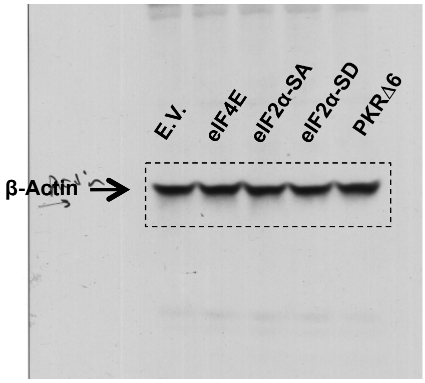

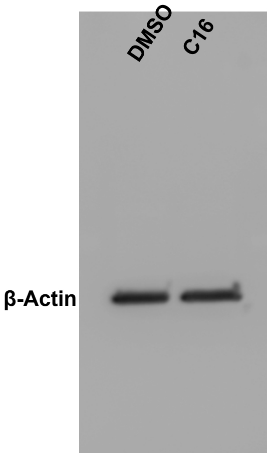

Full scans Figure 2e

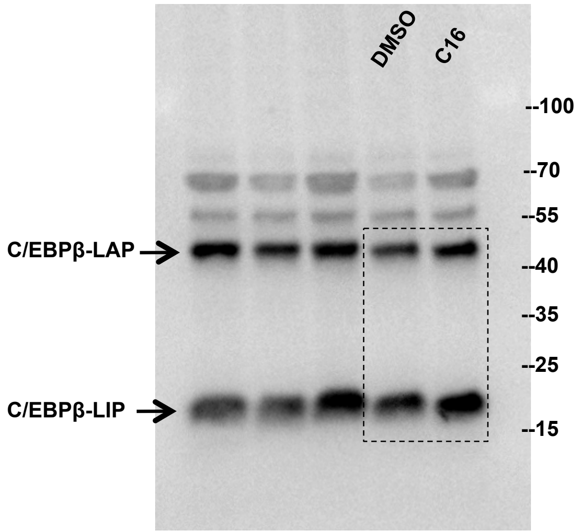

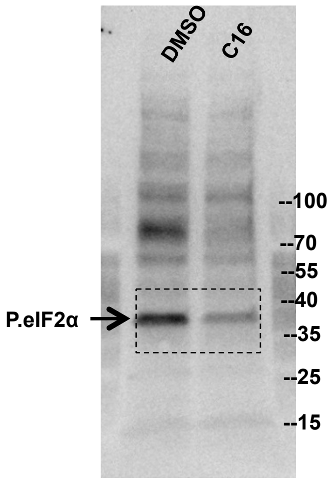

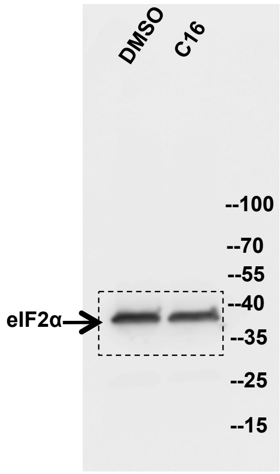

Figure S3

Full scans Figure 2f

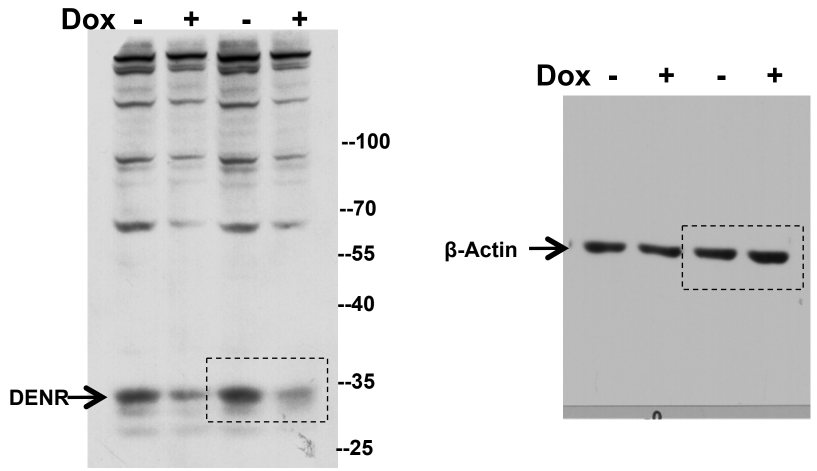

Full scans Figure 2g

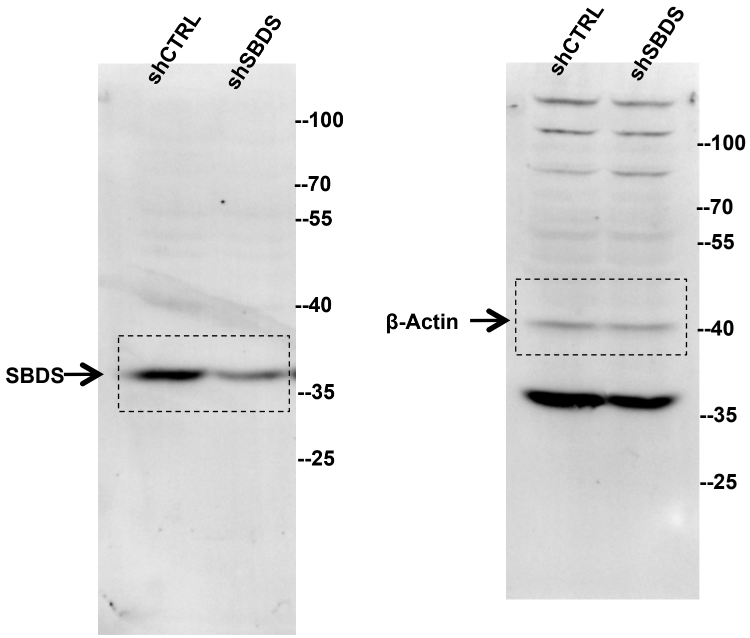

Figure S3. Full scans of Western blot data shown in Figure 2.
Rectangles delimit cropped areas used in the indicated panels in Figure 2.

Figure S4

Full scans Figure 4d

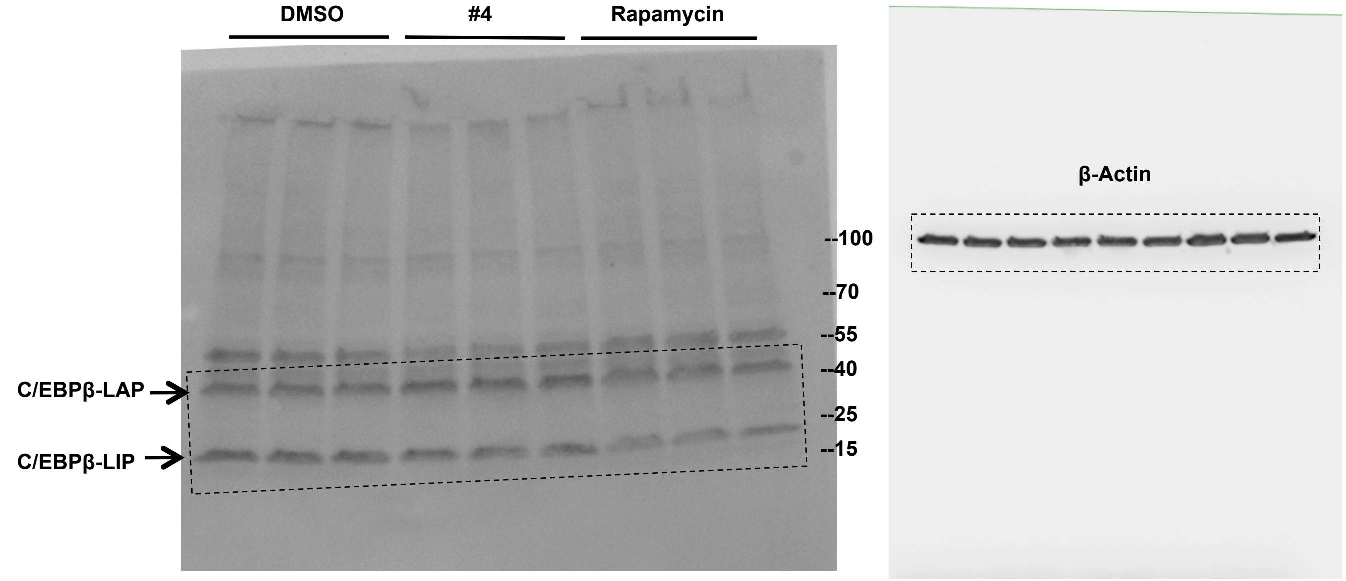

Full scans Figure 4e

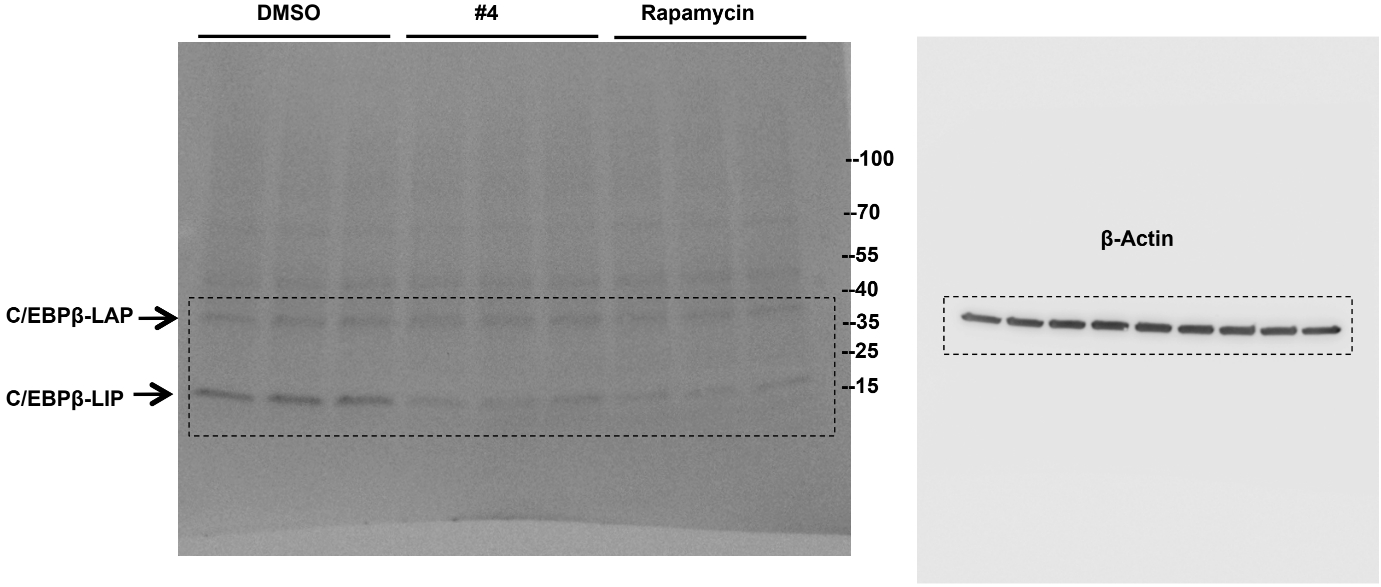

Figure S4

Full scans Figure 4f

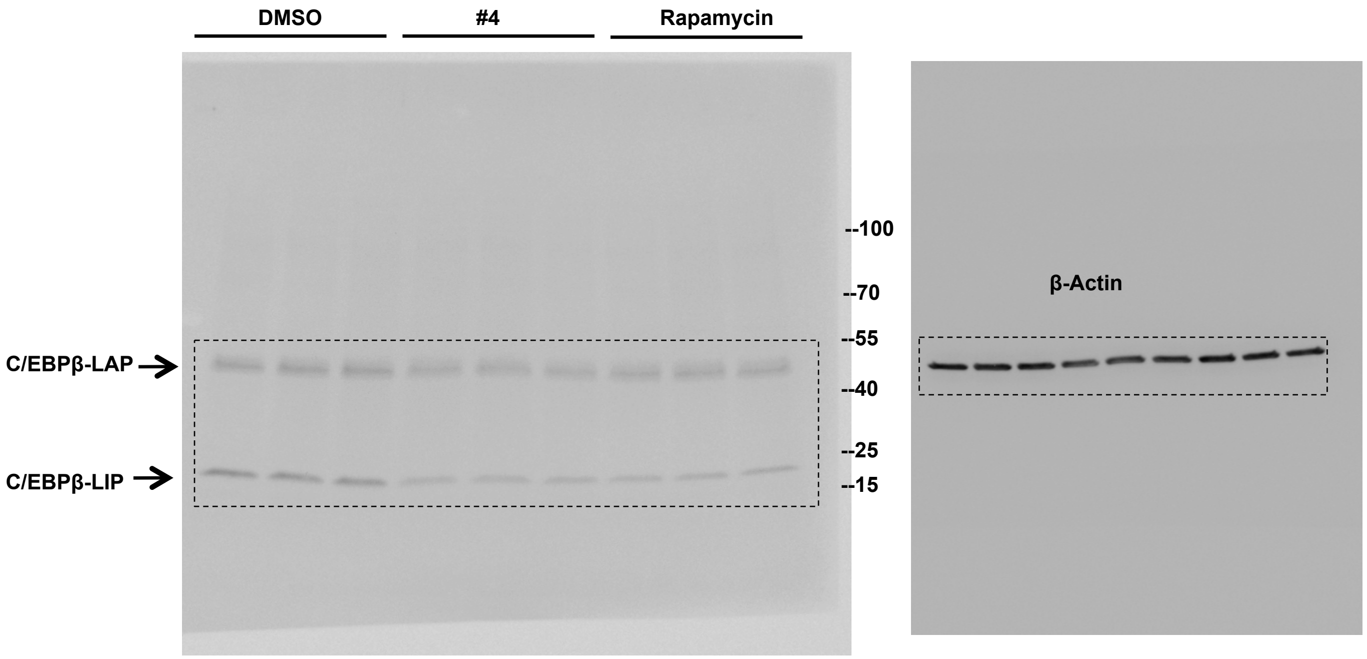

Figure S4. Full scans of Western blot data shown in Figure 4.
Rectangles delimit cropped areas used in the indicated panels in Figure 4.

Figure S5

Full scans Figure 5a

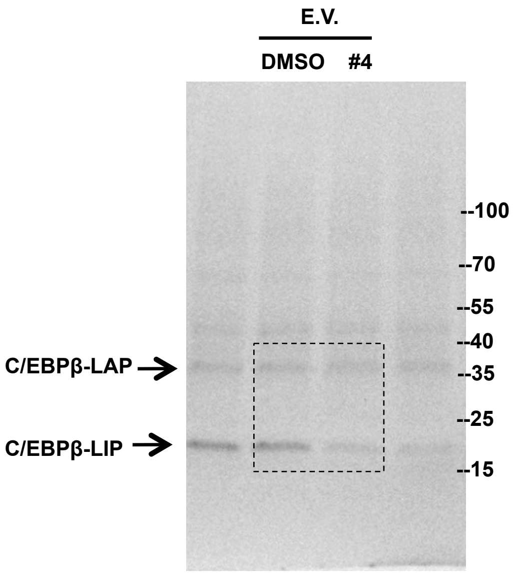

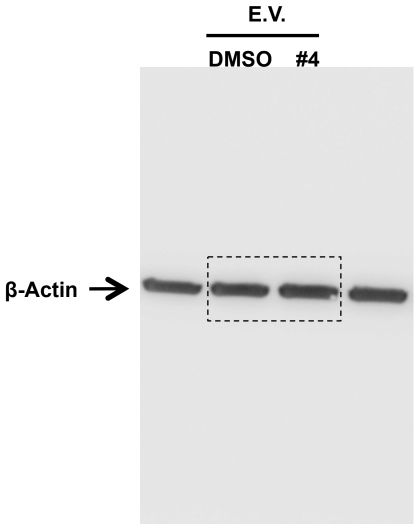

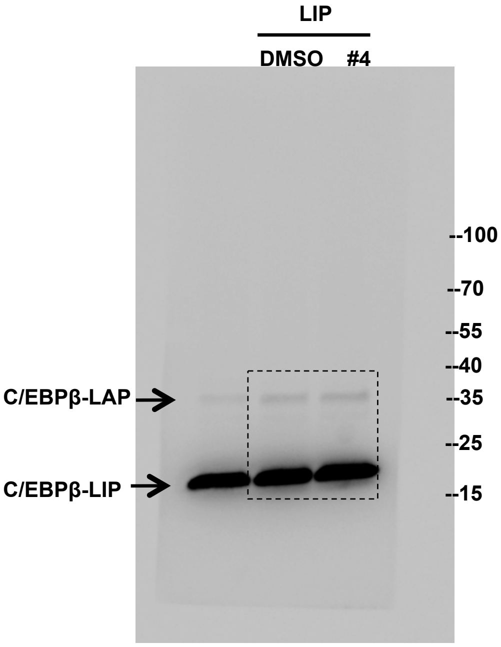

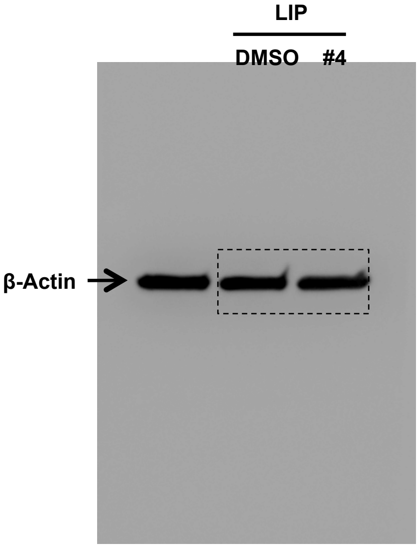

Figure S5. Full scans of Western blot data shown in Figure 5.
Rectangles delimit cropped areas used in the indicated panels in Figure 5.
